# Protein Persulfidation in Plants: Function and Mechanism

**DOI:** 10.3390/antiox10101631

**Published:** 2021-10-16

**Authors:** Peng Wang, Hua Fang, Rong Gao, Weibiao Liao

**Affiliations:** College of Horticulture, Gansu Agricultural University, 1 Yinmen Village, Anning District, Lanzhou 730070, China; pengsir0828@gmail.com (P.W.); Fanghua1610@163.com (H.F.); gaorong1005@gmail.com (R.G.)

**Keywords:** hydrogen sulfide, persulfidation, growth and development, antioxidant, phytohormone, autophagy, *S*-nitrosylation

## Abstract

As an endogenous gaseous transmitter, the function of hydrogen sulfide (H_2_S) has been extensively studied in plants. Once synthesized, H_2_S may be involved in almost all life processes of plants. Among them, a key route for H_2_S bioactivity occurs via protein persulfidation, in which process oxidizes cysteine thiol (R-SH) groups into persulfide (R-SSH) groups. This process is thought to underpin a myriad of cellular processes in plants linked to growth, development, stress responses, and phytohormone signaling. Multiple lines of emerging evidence suggest that this redox-based reversible post-translational modification can not only serve as a protective mechanism for H_2_S in oxidative stress, but also control a variety of biochemical processes through the allosteric effect of proteins. Here, we collate emerging evidence showing that H_2_S-mediated persulfidation modification involves some important biochemical processes such as growth and development, oxidative stress, phytohormone and autophagy. Additionally, the interaction between persulfidation and *S*-nitrosylation is also discussed. In this work, we provide beneficial clues for further exploration of the molecular mechanism and function of protein persulfidation in plants in the future.

## 1. Introduction

After translation, the regulation of protein function mainly rests on post-translational modifications (PTMs), protein-protein interactions, as well as the tight interplay between them. More than 400 different PTMs have been identified in eukaryotes to date [1]. Among them, the formation and regulation mechanism of some redox-based PTMs have been investigated in recent years, such as SUMOylation, phosphorylation, histone acetylation, methylation, ubiquitinylation, *S*-nitrosation, *S*-nitrosylation, and persulfidation. There is evidence that these modifications can change the activity of protein through allosteric effects and create binding domains that interacted with other proteins [2]. In addition, these PTMs have massively expanded the proteome, resulting in plethoric protein functions [3], to a certain extent, which breaks through the incarceration of genetics and genes on protein function.

At present, some important gas signal molecules are considered to exert their regulatory roles through PTMs, such as nitric oxide (NO) and hydrogen sulfide (H_2_S). In particular, ever since it was reported that there was a large amount of persulfidation protein in *Arabidopsis thaliana* [4], the reports about the regulation of plant life activities by persulfidation are springing up. Under normal growth conditions, Li et al. showed that the persulfidation of some root proteins in *A. thaliana* played a crucial role as a switch in its root growth and activity [5]. Some flowering-related transcription factors that were persulfidated advanced and extended the flowering period in Chinese cabbage [6]. Under adversity, since some antioxidant enzymes could undergo persulfidation modification, the tolerance of tomato seedlings to copper toxicity was enhanced [7]. During the cold stress test of *A. thaliana*, a number of key proteins of the stress response pathway were detected to be persulfidated to enhance low temperature tolerance [8]. Additionally, persulfidation modification has also been shown to play a key role in abscisic acid (ABA)-induced stomata closure or ethylene (ETH) pathway [9,10]. Thus, protein persulfidation has been well documented in scientific research as a regulating component of plant metabolism, and it has penetrated almost all aspects of plant life.

Although the research of protein persulfidation is surging in plants, it is very necessary to have a global outlook on the latest developments in this field. Within this context, this article systematically summarizes the roles of protein persulfidation in plant growth and development, stress response, and metabolic crosstalk, and comprehensively updates the latest research progress of persulfidation in plant systems.

## 2. H_2_S and Protein Persulfidation in Plants

H_2_S, a colorless lipophilic gas molecule, is notorious for its rotten egg smell. In nature, it was released predominantly by volcanic eruptions and geothermal events [11]. Over the last hundred years, H_2_S has always been consideration as a toxic substance for organisms [12,13], because high H_2_S concentration can inhibit the action of cytochrome c oxidase, which is the key enzyme for mitochondrial respiration, leading to the break of respiratory chain [14,15]. Doooej et al. suggested the positive effects of H_2_S on plants at micromolar concentrations [16]. As of late, H_2_S has been well-established as a gasotransmitter, which is similar to NO and carbon monoxide (CO). H_2_S in organisms is mainly produced by the metabolism of sulfur-containing amino acids. In animal cells, cystathionine γ-lyase (CSE) and cystathionine β-synthase (CBS) are the main enzymes responsible for H_2_S production, in which L-cysteine or L-homocysteine is used as a substrate (Table S1) [17]. Additionally, Mikami et al. found that thioredoxin (Trx) and dihydrolipoic acid (DHLA) associate with 3-mercaptopyruvate sulfur transferase (3-MST) to release H_2_S in mouse brains [18]. Meanwhile, H_2_S could also be degraded by Sulfide: quinone reductase, sulfur dioxygenase and thiosulfate: cyanide sulfur-transferase in mammalian cells. Moreover, the thiol S-methyltransferase in cytosol can methylate H_2_S to form methanethiol and dimethyl sulfide [17]. Importantly, previous studies suggested that H_2_S could also generate endogenously in plants through multiple enzymatic pathways [19,20,21]. Desulfhydrase L-Cys (L-CD) is the animal equivalent CSE enzyme which is responsible for the cytoplasm H_2_S production in plant cells [22]. In addition, D-cysteine desulfhydrase (D-CD) and cyano alanine synthase (CAS) are H_2_S producing enzymes located in the mitochondria. Chloroplast may also be a source of H_2_S, in which H_2_S is generated through the action of sulfite reductase (SiR) [22]. However, due to the alkaline pH in cell compartments, the H_2_S formed within chloroplasts and mitochondria probably may not diffuse into the cytoplasm [23]. Hence, desulfhydrase activity in the cytoplasm and H_2_S in cytosolic pool may function as a signaling molecule. Interestingly, DHLA is also present in plant cells, which is a physiological substance corresponding to dithiothreitol (DTT) [24]. Until now, it is unclear whether DHLA is the source of H_2_S in plant cells. The level of H_2_S in cells is the result of not only its biosynthesis, but also its degradation. O-acetylserine-thiol lyases (OAS-TL), also known as cysteine synthase (CS), could reduce the concentration of cytoplasmic H_2_S by synthesizing cysteine [25]. Notably, the reverse reaction catalyzed by OAS-TL could also degrade cysteine to produce H_2_S (Table S1). H_2_S is exclusively or partly involved in many major biochemical processes of plants, such as seed germination [26], root organogenesis [27], photosynthesis [28], stomatal aperture [29], plant senescence [30] and post-harvest storage processes [31,32]. H_2_S is also associated with antioxidant mechanisms [33,34], autophagy [35], and various anti-stress processes [36,37]. Although a large number of studies have confirmed that H_2_S is closely related to plant life, the potential mechanism of H_2_S as a signal molecule in plants is being explored.

Signal molecules usually perform their cellular functions by binding to receptors. There has been increasing attention to how cells perceive H_2_S signaling. In recent years, a novel H_2_S-mediated protein reversible redox-based PTM has been deciphered. This process was designated as protein persulfidation (previously known as S-sulfhydration). Protein persulfidation may be the main pathway by which H_2_S acts as a signaling molecule in organisms [38]. Persulfidation occurs on cysteine thiols of target proteins, which oxidizes cysteine thiol (R-SH) groups into persulfide (R-SSH) groups [39]. Accordingly, the conformation of some proteins has changed by this modification, thus resulting change of target protein functions. Cuevasanta et al. confirmed that persulfides had higher nucleophilic reactivity in comparison to parent thiol groups [40]. Therefore, the reactivity of the modified protein is usually altered. For example, Aroca et al. reported that the activity of glyceraldehyde 3-phosphate dehydrogenase (GAPDH) and ascorbate peroxidase (APX1), which modified by persulfidation in *A. thaliana* was increased [4]. Conversely, the glutamine synthetase (GS2) was inactivated through this modification. However, these changes can be reversed by adding dithiothreitol (DTT), which is disulfide bond synthesis inhibitor [4]. There was also evidence suggested that protein persulfidation affected subcellular localizations of target proteins. For example, the nuclear localization of GAPDH in *A. thaliana* was enhanced by persulfidation [41].

The biochemical process of protein persulfidation is always controversial in organisms. Because from the view of thermodynamics, H_2_S could not react directly with thiols of cysteine [38], which implied that H_2_S-induced protein persulfidation might involve a variety of intricate biochemical process. On one hand, persulfides (RSSH) are formed as a result of oxidation of thiols (RSH). It means that sulfur changes its oxidation state from −2 to −1, and therefore persulfidation cannot be the result of direct reaction of thiol residues with H_2_S. In this reaction, the compounds containing sulfane sulfur, which is at a −1 or 0 oxidation state, play the role of oxidizing agents [4,42,43]. On the other hand, only if the thiol group is reversibly oxidized to the form of sulfenic acid (-SOH), *S*-nitrosothiol (-SNO) or disulfide (-SSR), the HS^-^ anion resulting from dissociation H_2_S can react with these oxidized thiol group to form RSSH [44,45,46]. Currently, five possible mechanisms by which protein persulfidation can occur have been proposed in cells. First, *S*-sulfenylation is a reversible oxidative PTM of cysteine thiols, which oxidizes thiols of cysteine into sulfenic acid (R-SOH) groups. Reyes et al. used peroxiredoxin alkyl hydroperoxide reductase E of *Mycobacterium tuberculosis* (MtAhpE-S-) as the research material to confirm that H_2_S could react with MtAhpE-SOH (MtAhpE-S- of *S*-sulfenylation) to form persulfides (MtAhpE-SS-) [47]. It has also reported that the GAPDH of *S*-sulfenylation could further react with H_2_S to form persulfides in mammals [48]. Therefore, the reaction between H_2_S and R-SOH is one of the ways of protein persulfidation. Second, *S*-nitrosylation is an important PTM of protein, which oxidates cysteine thiols into *S*-nitrosothiol (R-SNO). It was reported that H_2_S could react with R-SNO to form thionitrous acid (HSNO) in humans [49]. However, HSNO had high activity and was extremely unstable so it might further be oxidized by H_2_S to form persulfides [50]. Filipovic et al. also speculated that due to the influence of the cell environment of cysteine on the thiol groups [49], H_2_S might directly react with R-SNO to form persulfides in humans. Third, under normal circumstances, the reaction of thiols and disulfides (RSSR) in cells might maintain dynamic equilibrium. Cuevasanta et al. suggested that H_2_S could react with low molecular weight (LMW) RSSR and mix protein RSSR to produce persulfides in mammals [40], suggesting that the reaction of H_2_S with RSSR is also a source of persulfides in cells. Fourth, except for the reaction between H_2_S and the oxidized thiol derivatives, the reaction of polysulfides (HSx^•−^) with cysteine thiols have also been shown to be a way to generate persulfides. All sulfur atoms in polysulfides (HSx^•−^) could be attacked by free cysteine thiols to form persulfides [43]. Finally, the formation of protein persulfidation may also involve metal centers. H_2_S could be oxidized by some iron haem centres to HS^•−^ [38], which could further react with free cysteine thiols to generate protein persulfides [48]. Likewise, Zhang et al. reported that with the presence of iron porphyrin [48], GAPDH and H_2_S could generate protein persulfides. Although there above mechanisms are reasonable, in the specific biochemical reaction, which one of the above mechanisms works is still uncertain.

## 3. Roles of Protein Persulfidation in Plant Growth and Development

At present, the emerging evidence suggests that protein persulfidation is an important regulatory component during plant growth and development. For the first time, Aroca et al. detected that there were 106 proteins undergo persulfidation in *A. thaliana* leaves by the improved biotin switch method and liquid chromatography coupled to mass spectrometry (LC-MS/MS) analysis [4]. However, due to the low specificity of this method for thiols groups and persulfides groups, the results have been questioned. A new tag-switch assay revealed that 2015 proteins of *A. thaliana* were persulfidated, suggested at least 5% whole *A. thaliana* proteome may be persulfidated under free-stress conditions [51]. The authors further investigated the functional classification of detected proteins through MapMan classification and Gene Ontology (GO) enrichment analysis. Out of 367 persulfidated proteins in *A. thaliana* leaves, 25 distributed in subcellular targeting, 38 distributed in PTM, 38 distributed in glycosylation and assembly, 79 distributed in protein synthesis, and 144 distributed in degradation and folding [51]. In addition, data in that report demonstrated that multiple enzyme systems and amino acid metabolism were also regulated by protein persulfidation. Based on *A. thaliana* proteins reference database, GO enrichment result showed that 30 out of 57 glycolysis proteins, 41 out of 84 tRNA aminoacylation proteins, and 214 out of 1471 abiotic stress-related proteins have been persulfidated. The above results indicate that protein persulfidation plays an important role in plant primary metabolism (including the Calvin cycle, glycolysis, and the tricarboxylic acid cycle), plant growth and development, RNA transcription and plant tolerance to abiotic stress. Obviously, from the result of proteomics analysis, protein persulfidation is involved in a variety of processes in plant life activities. To further illustrate the regulatory mechanism and important biological functions of these candidate proteins in plants, specific methods are needed rather than proteomic analysis.

**Figure 1:**
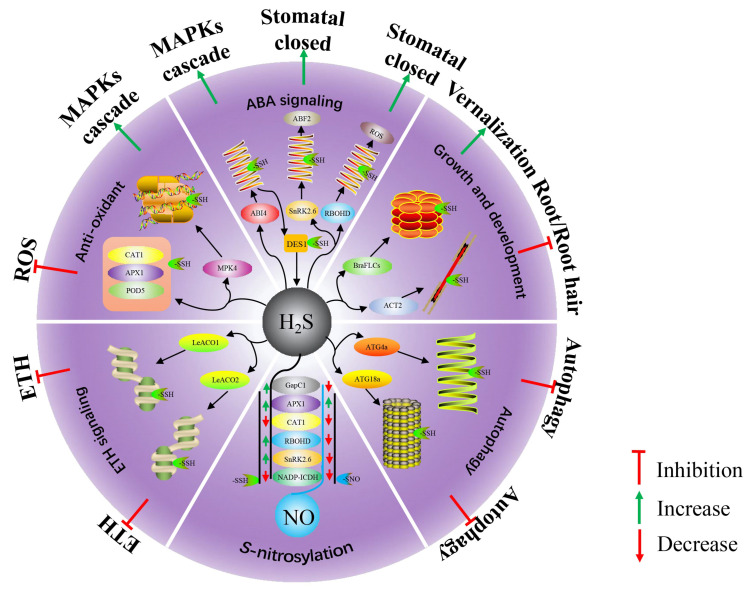
Hydrogen sulfide (H_2_S) regulates the molecular mechanism of a series of physiological and biochemical processes in the plant system. Integrative schematic representatives the regulatory mechanism of H_2_S-induced protein persulfidation in plant systems. A major route for H_2_S bioactivity is through protein persulfidation (-SSH) to form persulfidated proteins. H_2_S-modified regulators function in diverse metabolic process and signaling pathways, including anti-oxidant, growth and development, autophagy, abscisic acid (ABA) signaling, ethylene (ETH) signaling, and interaction with *S*-nitrosylation (-SNO). Plant functions regulated by these processes are indicated at the periphery of the diagram. DES1, desulfhydrase 1; ABI4, abscisic acid insensitive 4; SnRK2.6, SNF1-RELATED PROTEIN KINASE2.6; RBOHD, respiratory burst oxidase homolog protein D; ABF2, ABA response element-binding factor2; ROS, reactive oxygen species; ACT2, actin2; BraFLCs, Chinese cabbage Flowering Locus C; ATG, autophagy-related proteins; LeACO1/ LeACO 2, ACC oxidases; CAT1, catalase 1; APX1, ascorbate peroxidase 1; POD5, peroxidase 5; MPK/MAPK, mitogen-activated protein kinases.

The involvement of H_2_S in plant root organogenesis has been known for some time, but the underlying mechanism has been explored in recent years. Mei et al. found that pretreatment with 2 mM Na_2_S (a donor of H_2_S) could enhance the level of persulfidation in tomato root protein extract [52]. While DTT was applied, the above effects were diminished or disappeared. When the homeostasis of endogenous H_2_S was destroyed, the persulfidation level of the proteins also changed in response [52]. Of note, this change is consistent with the lateral root phenotype of tomato seedlings. But the specific mechanism that H_2_S regulates the formation of tomato lateral roots through protein persulfidation is not yet clear. Furthermore, H_2_S is also considered to play a role in root hair formation. Li et al. found that the levels of protein persulfidation in H_2_S-overproducing *A. thaliana* mutants and transgenic plants were significantly increased [5]. They further found that persulfidation was located on Cys287 of ACTIN2 (ACT2), a member of plant actin groups. Therefore, two transgenic lines plants, *act2-1/ACT2^C287S^* and *act2-1/ACT2^WT^* were used to explore the signal function of H_2_S. The absence function of *act2-1* in *act2-1/ACT2^C287S^* was partly restored and completely restored in *act2-1/ACT2^WT^*. Notably, in the presence of NaHS (another donor of H_2_S), root hair growth and root growth rate were suppressed in *act2-1/ACT2^WT^* plants, yet they were not affected in *act2-1/ACT2^C287S^* plants, suggesting Cys287 of ACT2 might be a target of persulfidation and H_2_S inhibited root hair growth of *A*. *thaliana* by regulating the level of persulfidation of ACT2 protein. In addition, H_2_S might play an important role in plant flowering. Very recently, Ma et al. showed that exogenous application of 100d μM H_2_S not only advanced the flowering period of Chinese cabbage, but also prolonged the flowering period [6]. Subsequently, they found that some transcription factors from Chinese cabbage Flowering Locus C (BraFLCs) had undergone persulfidation modification. These transcription factors controlled vernalization by combining with Suppressor of Overexpression of Constant 1 (SOC I). Corresponding probe detection found that this modification blocked the binding of BraFLCs to downstream promoters *SOC I*. Thus, an important mechanism that H_2_S regulate the flowering of plants by causing member of BraFLCs persulfidation to compensate insufficient vernalization was deciphered. Collectively, H_2_S can change the persulfidation level of some proteins to regulate plant growth and development (Figure 1).

## 4. Roles of Protein Persulfidation in Plant Abiotic Stress

### 4.1. Antioxidant Protection Mechanism

**Figure 2:**
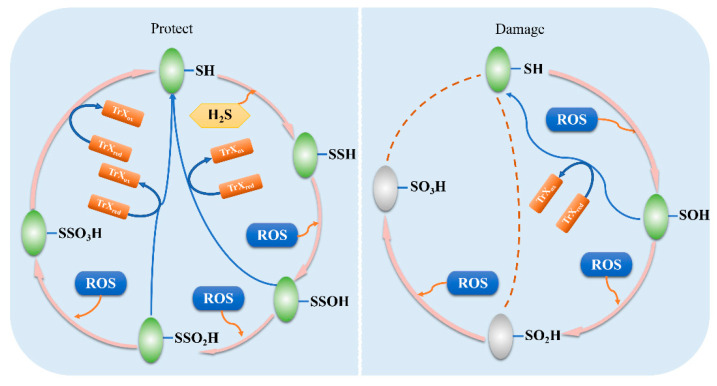
The protection mechanism of protein persulfidation. Under stress conditions, the accumulation of H_2_S leads to the persulfidation (-SSH) of downstream proteins. R-SSH can also be oxidized by high level reactive oxygen species to perthiosulfenic acids (R-SSOH), further oxidation may form perthiosulfinic (R-SSO_2_H) or perthiosulfonic (R-SSO_3_H) acids. This can be rescued by thioredoxin (Trx_red/ox_), which restores the original function of the protein. Thus, it creates a reversible redox protection mechanism for protein cysteine. When H_2_S is absent, the accumulation of ROS leads to sulfenic acid (R-SOH) of downstream proteins. R-SOH can also be further oxidized to sulfinic (R-SO_2_H) or sulfonic (R-SO_3_H) acids. Normally, R-SO_2_H and R-SO_3_H cannot be reversed, leading to irreversibly changes in protein functions even inactivation. This causes irreversible oxidative damage to protein cysteine.

Unlike animals, plants grow in a fixed position without motor function and nervous system, thus suffering a variety of environmental stresses more easily, including biotic stress and abiotic stress. Abiotic stresses are usually accompanied by the accumulation of reactive oxygen species (ROS) and reactive nitrogen species (RNS) in plants. Among them, ROS includes super-oxide anion radicals (O_2_^•−^), hydrogen peroxide (H_2_O_2_), hydroxyl radicals (OH^•−^), and singlet oxygen (^1^O_2_). Within the normal threshold, ROS play an essential role in variety signaling pathways as a secondary messenger [53]. However, ROS excessive accumulation can cause oxidative stress and may result in irreversible damage to cells [54]. Although the corresponding antioxidant systems are activated by these stresses to maintain the balance of ROS in plants, the scavenging power of these antioxidant systems to remove ROS is limited. Under persistent oxidation conditions, R-SOH can be further oxide to sulfinic (R-SO_2_H) or sulfonic (R-SO_3_H) acids. Normally, R-SO_2_H and R-SO_3_H cannot be reversed, leading to irreversibly changes in protein functions even inactivation [55]. R-SSH can also be oxidized by high level ROS to perthiosulfenic acids (R-SSOH), further oxidation may formation perthiosulfinic (R-SSO_2_H) or perthiosulfonic (R-SSO_3_H) acids, which can reduce the level of ROS in the cell. More importantly, all of these oxidized forms can be rescued into thiols to restore the original function of the protein [38]. Therefore, the conversion between R-SH and R-SSH of cysteine acts as a way to exert protective roles in plants under stresses by on-off switching protein functions and reducing ROS levels in cells, which effectively mitigates the oxidative damage and enhances the tolerance of plants under adverse conditions (Figure 2).

Antioxidant enzymes, such as ascorbate peroxidase (APX), superoxide dismutase (SOD), peroxidase (POD), and catalase (CAT), play a key role in maintaining the balance of ROS in plants. In recent years, an increasing number of reports indicated that H_2_S can enhance the activity of antioxidant enzymes to respond abiotic stresses. For example, exogenous application of 2 µM NaHS could significantly change APX, SOD, POD, and CAT activities in rice plants, and effectively alleviate the damages of aluminum toxicity [56]. The same result was also confirmed in cucumbers with excessive nitrogen or chilling damage [57,58]. Recently, the mechanism about how H_2_S changes the activity of antioxidant enzymes was answered by Li et al. [7], who demonstrated that H_2_S modified the activity of antioxidant enzymes through persulfidation. They found that the growth of the above-ground part and root system of tomato seedlings treated with high concentration of copper oxide (II) nanoparticles (CuO NPs) were significantly inhibited. The level of ROS was significantly increased and content of malondialdehyde (MDA) which is an indicator reflecting the degree of membrane peroxidation also increased in tomato seedlings under CuO NPs stress [7]. However, after treatment with 200 µM NaHS, this membrane oxidation damage caused by CuO NPs showed a significant alleviation. Notably, both in vivo and in vitro tests showed that after NaHS treatment, SOD activity did not change significantly; CAT activity decreased slightly, while APX and POD activity increased. The authors used recombinant protein SlCAT1, SlcAPX1 and SlPOD5 further found that these enzymes could be persulfidated by NaHS in a dose-dependent manner. In SlCAT1, Cys234 might be a persulfidation site close to the enzyme active domain. Cys168 located in the active domain of SlcAPX1 could also undergo persulfidation modification. Moreover, both Cys46 and Cys61 in the active domain of SlPOD5 could be persulfidated. Thus, under oxidative stress, H_2_S could increase the activity of APX and POD by inducing persulfidation (Table 1).

The study of Aroca et al. also showed that the APX activity after persulfidation was enhanced [4]. Li et al. reported that after persulfidation modification the activity of CAT was inhibited [7], which is consistent with results of Corpas et al. [20]. Although NaHS could maintain SOD activity under CuO NPs stress, no persulfidation sites had been found in SOD, so the regulation of SOD activity by H_2_S may involve other mechanisms at the transcriptional level [7]. Therefore, H_2_S-mediated persulfidation can maintain the level of ROS under abiotic stress by changing the activity of some antioxidant enzymes, thereby enhancing the stress resistance of plants (Figure 1).

Apart from the persulfidation modification of antioxidant enzymes in response to abiotic stress, mitogen-activated protein kinases (MAPKs) might play an important role in alleviating adversities in plants. MAPKs involve a cascade of reactions MEKK → MEK → MPK for signal transduction [62]. Previously, Teige et al. found that MEK1-MEK2-MPK4 was an important pathway in *A. thaliana* in response to salt and cold stresses [63]. Intriguingly, MPK4 has a close relationship with H_2_S. Du et al. reported that in *A. thaliana* under cold stress, MPK4 acted as a downstream signal of H_2_S to respond to cold damage by regulating cold response-related genes and stomatal movement [64]. To further investigate how H_2_S regulates MAPKs cascade to alleviate cold damage, using *crlk1*, *mek2* and *mpk4* mutants, Du et al. found that 100 μM NaHS could alleviate the damage caused by low temperature to *crlk1* and *mek2* mutants, but did not alleviate the effect on *mpk4* [8]. The biotin-switch method showed that H_2_S increased the persulfidation level of MPK4, and the activity of MPK4 was significantly improved after H_2_S pretreatment. The above results indicate that H_2_S may increase the activity of MPK4 by regulating the persulfidation level of MPK4 and ultimately respond to cold stress (Table 1). Taken together, H_2_S can regulate the persulfidation level of some key proteins in abiotic stress response, resulting in the enhancement of some stress response signal cascades, thereby enhancing the tolerance to abiotic stress (Figure 1).

### 4.2. Protein Persulfidation in Phytohormone Signal

Phytohormones are the most important group of endogenous signal molecules in plants, which ubiquitously regulates growth and development processes and stress responses, ranging from seed germination to plant senescence. Phytohormones include ABA, ETH, gibberellin (GA), auxin (AUX), and cytokinin (CTK) etc. Among them, ABA plays a pivotal role in the response and adaptation of plants to a variety of unfavorable environments. Stomatal movement is another important physiological indicator of plants to respond to abiotic stress. Especially under drought stress, plants will reduce water loss by adjusting the stomata aperture [65]. Previous studies have shown that ABA is important signal molecules in the process of regulating stomatal movement [66,67]. Importantly, the role of L-CYSTEINE DESULFHYDRASE 1 (DES1) which is the main enzyme for H_2_S production has also been reported in stomatal movement in recent years [68]. Therefore, H_2_S and ABA may interact in the process of regulating the opening and closing of stomata. As expected, Aroca et al. reported that the persulfidation level of ABA receptors PYRABACTIN RESISTANCE 1 (PYR1) and PYR1-LIKE PROTEIN 1 (PYL1) has altered in *des1*, a DES1 loss-of-function mutant, and ABA loses the ability to induce stomata closure [51]. To confirm the interaction between ABA and H_2_S in stomatal movement, Zhang et al. used *A. thaliana aba3* mutants deficient in ABA synthesis, *des1*, and *aba3*/*des1* double mutants for verification [69]. In *aba3* mutants, dehydration-induced *DES1* expression was abolished, while restored when exogenous ABA treatment or *aba3* expression. Correspondingly, in *des1* mutants, the drought-induced ABA synthesis gene expression was suppressed. Only when NaHS was used exogenously or *aba3* and *des1* were expressed simultaneously in *aba3*/*des1* double mutants, the wild-type phenotype could be completely restored. Thus, under drought stress, H_2_S and ABA synergistically regulate stomatal movement, and both are indispensable.

Although a small percentage were report on the mechanism by which H_2_S plays a role in ABA-mediated stomata movement, some important advances have been made. A recent study reported that ABA could stimulate DES1 to produce H_2_S in *A. thaliana* guard cells [68]. Soon, Shen et al. revealed that ABA induced persulfidation modification of Cys44 and Cys205 sites of DES1 in a redox-dependent manner (Table 1), which promoted the release of H_2_S from DES1 [59]. Calcium ion (Ca^2+^) is the most direct regulator of stomata movement that can close the stomata by regulating the efflux of other ions and forcing the guard cells to lose water [70]. SNF1-RELATED PROTEIN KINASE2.6 (SnRK2.6), also known as Open Stomata 1 (OST1), could be activated through ABA and acted as an upstream signal of the cytosolic Ca^2+^ [71]. Chen et al. found two persulfidation sites Cys131 and Cys137 on the surface of SnRK2.6 (Table 1) and confirmed that H_2_S dependent on DES1 could cause the persulfidation modification of SnRK2.6 [68]. In vitro tests proved that the activity of SnRK2.6 modified by persulfidation was significantly increased [68]. Of note, the interaction between the persulfidation-modified SnRK2.6 and ABA RESPONSE ELEMENT-BINDING FACTOR2 (ABF2) has been strengthened, which formed a positive regulatory loop and promoted ABA signaling. Physiologically, ABA-induced stomatal closure was enhanced when the Cys131 and/or Cys137 sites of SnRK2.6 were persulfidated [68]. The authors further found that the Ca^2+^ influx in the Cys131 and Cys137 site mutants was significantly reduced compared with the wild type, which means that the sensitivity of the stomata to ABA signals was reduced. In other words, under drought conditions, ABA cannot induce stomata closure in time to reduce water loss, which reduces the drought tolerance of the plant. This mechanism is an important breakthrough in H_2_S-mediated ABA-induced stomatal closure. In addition to SnRK2.6, NADPH oxidases respiratory burst oxidase homolog protein D (RBOHD) is a key enzyme responsible for the production of ROS in plants. Shen et al. revealed the roles of RBOHD in ABA signaling and demonstrated DES1-dependent H_2_S accumulation could induce RBOHD to undergo persulfidation modification at the Cys825 and Cys890, which improved the ability of RBOHD to generate ROS (Table 1) [59]. Consistent with mentioned earlier, the persulfidated DES1 and RBOHD were further oxidized by the accumulated ROS and the effective function of the DES1 and RBOHD were temporarily suppression or loss, resulting in the de-sensitization of DES1 to ABA. Meanwhile, Shen et al. found when the Cys825 and Cys890 of RBOHD were replaced by alanine, the ABA-induced stomatal closure was abolished [59]. Therefore, H_2_S could negatively regulate ABA signaling through persulfidation (Figure 1).

ABSCISIC ACID INSENSITIVE 4 (ABI4) is a multifunctional transcription factor and studies showed that it was involved in ABA signaling [72]. ABI4 plays a documented role in the expression of stress-responsive genes. It can bind to coupling element 1 (CE1)—a fragment of special motif CACCG—within their promoters to regulate the expression of target genes [56]. In addition, the model established by Wind et al. showed that ABI4 might also be combined with G-box (CACGTG) [73]. Remarkably, When ABI4 binds to CE1, the ability of G-box to bind other transcription factors is abolished. Therefore, CE1 may have overlapping fragments with G-box, which has been confirmed in hybrid yeast experiments [74]. Since both DES1 and ABI4 are involved in ABA-induced stomatal closure, Zhou et al. showed that ABI4, as a downstream signal of DES1 or H_2_S, played a role in stomatal closure [9]. Based on this, Zhou et al. analyzed the persulfidation level of ABI4 and both in vivo and in vitro tests have confirmed that Cys250 of ABI4 could be persulfidated [9] (Table 1). Likewise, among the three predicted persulfidation sites Cys180, Cys250, and Cys283, only when Cys250 was mutated, ABA-induced stomatal closure was hyposensitized. However, Cys180 and Cys283 mutations had no effect on ABA and H_2_S signals, indicating that Cys250 residues of ABI4 might be a key factor in ABI4 response to ABA signaling. According to Bai et al., ABI4 was regulated by the MAPK signaling cascade in the process of *A. thaliana* adventitious root formation [75]. Mitogen-Activated Protein Kinase Kinase Kinase 18 (MAPKKK18), a member of the MEKK family, was confirmed to play a role in ABA signaling in *A. thaliana* [76]. The promoter of MAPKKK18 contains the CE1 element, which illustrated that ABI4 might have the potential to directly bind to MAPKKK. To further insight the interaction between ABI4 and MAPKKK18, Zhou et al. used the yeast one-hybrid (Y1H) assay, and the results showed that ABI4 could specifically bind to the CE1 of the MPKKK18 promoter to activate the transcription of MPKKK18 [9]. Notably, the Cys250 residue mutant line of ABI4 cannot bind to MAPKKK18, which indicates that ABI4 Cys250 determines the binding of ABI4 to MAPKKK18. More importantly, Zhou et al. showed that, in ABA signaling, DES1/H_2_S could promote the transcription of MAPKKK18 through the persulfidation modification of ABI4 Cys250, but this modification did not promote the binding of ABI4 and MAPKKK18 [9]. In addition, ABI4 could also bind to the promoter of *DES1*, which formed a positive regulatory loop to promote MAPK cascade (Figure 1). Therefore, these studies deciphered the mechanism of H_2_S-mediated protein persulfidation in ABA signaling. When plants respond to adversities, especially osmotic stress, H_2_S promotes or inhibits ABA signaling by persulfidation modifying one or more key proteins in the process of ABA signaling. Physiologically, the ABA signal can control the opening and closing of the stomata in response to adversities.

ETH, as a gaseous phytohormone, plays an important role in plant growth, fruit ripening, and stress response. Within the normal threshold, ETH can enhance plant resistance to adversity conditions. However, excessive accumulation will accelerate plant senescence and lead to programmed cell death. In plants, ACC oxidases (ACOs) and 1-aminocyclopropane-1-carboxylic acid (ACC) synthases (ACSs) are two key enzymes in ETH biosynthesis, which accelerate the release of ETH in plants response to adversity. Tomato ACOs are encoded by a multi-gene family, including *LeACO1* and *LeACO2* and *LeACO3* [77]. Among them, *LeACO1* and *LeACO2* have high expression specificity in guard cells [78], which means that ETH has the potential to regulate stomatal movement. Notably, Xiao et al. showed that exogenous application 0.2 mM H_2_S could inhibit ETH synthesis caused by waterlogging in peach seedlings [79]. However, there is no further insight into the interaction mechanism of H_2_S and ETH in this study. In another study, Jia et al. reported that ETH induced H_2_S production in tomato guard cells and found that H_2_S could control ETH-induced stomatal closure under osmotic stress [10]. Further, the author revealed that H_2_S could cause persulfidation of LeACO1 and LeACO2 (Table 1), thereby reducing the enzyme activity of LeACO1 and LeACO2, which inhibits the synthesis of ETH in tomato. Clearly, a feedback regulation mechanism has been revealed, which enhances plant stress tolerance by inhibiting the over-synthesis of ETH (Figure 1). In addition, The Cys60 residue of LeACO1 has been confirmed to be a site where LeACO1 undergoes persulfidation modification, which is directly related to the function of LeACO1.

### 4.3. Protein Persulfidation in Plant Autophagy

Autophagy is a relatively conservative material circulation process in organisms. In plants, autophagy is involved in many aspects of plant life, including growth and development, immune response, and senescence [80]. It usually maintained a basal level under normal conditions to degrade damaged cell components and was induced to high levels during stresses, including drought, high temperature, low temperature, high salt stress, and hypoxia etc. [81]. Furthermore, autophagy is essential for nutrient allocation and balance in plants. There are at least three types of autophagy—macroautophagy, microautophagy, and Mega-autophagy in plant cells [81]. Macroautophagy (hereafter termed autophagy) is the main pathway for the degradation of cell contents. When autophagy was activated, autophagosomes with a double-layer membrane structure were formed, and then the autophagosomes package and transport damaged organelles or pathogens, which were degraded after fusion with lysosomes/vacuoles [82]. While microautophagy is a process in which pathogens or micro-cellular components are engulfed and degraded by the local invagination or protrusion of lysosomes/vacuoles membrane [83,84]. Usually, Mega-autophagy is an extremely bad event for plants, which is closely related to programmed cell death (PCD) and causes large-scale degradation of plant cells [85]. In these ways, the degradation of intracellular pathogens and the re-use of nutrients are realized. However, although some studies have reported some genes and proteins related to autophagy, the exact regulation mechanism of cells on autophagy is not yet clear.

Accumulated evidence indicates that more than 40 autophagy-related proteins (ATGs) have been identified. These proteins have a high degree of homology in animals, yeast and plants [80]. ATGs tightly regulate the process of autophagy and participate in the formation and release of autophagosomes, the fusion and enlargement of phospholipid membranes, and the size and number of autophagosomes [61,86,87]. Importantly, H_2_S might play an essential regulatory role in plant autophagy. In *A. thaliana,* DES1 deficiency or *des1* null mutants, ATG8 protein accumulation and lipidation were enhanced, which was a landmark of autophagy activation in plants [88]. In addition, DES1 deficiency showed autophagy-induction phenotypes like premature senescence and hypersensitivity to stress [88]. In other words, H_2_S can inhibit the activation of autophagy by suppressing the activity of ATG8 protein in *A. thaliana*. Likewise, Laureano-Marín et al. showed that H_2_S regulated autophagy in *A. thaliana* roots through a pathway independent of ROS under nitrogen deprivation [60]. However, the regulatory mechanism has always been controversial. Very recently, a label-free quantitative proteomic analysis revealed that some autophagosome formation-related proteins were prone to be persulfidated, such as ATG3, ATG5, ATG7, etc. Furthermore, some proteins of initial regulatory steps for autophagy could also be persulfidated [89]. Generally, in bulk autophagy, the binding of ATG8 to phospholipid phosphatidylethanolamine (PE) is a key step in the formation of autophagosomes. The Cys-type protease ATG4 plays an important role in this process. It can recognize and cleave the C-terminal extension sequence of ATG8 until the Gly that specifically binds to PE is exposed. Remarkably, a specific study showed that under conditions of autophagy-activating, such as nitrogen starvation or osmotic stress, the protease activity of AtATG4a was significantly increased, and 200 μM NaHS effectively reversed this phenomenon [90]. More importantly, the authors revealed that whether under basal conditions or autophagy-activating conditions, H_2_S could specifically modify the Cys170 residue of AtATG4a to cause reversible persulfidation (Table 1). The activity of persulfidated AtATG4a decreased, which weakened the catalytic effect of ATG8 and PE conjunction, thereby inhibiting the occurrence of autophagy [90]. Moreover, endoplasmic reticulum (ER) autophagy, also known as reticulophagy or ER-phagy, was a selective autophagy caused by ER stress and is tightly regulated by the ATG18a protein in *A. thaliana* [91]. Consistent with the conclusion of Laureano-Marín et al. [90], Aroca et al. reported that, under ER stress, the reversible persulfidation modification of ATG18a residue Cys103 enhanced its affinity and co-localization time with phagophore membranes (Table 1), thus delaying the release and maturation of autophagosomes [61]. However, when persulfidation was abolished, the size and number of autophagosomes were affected [61].

In short, in plants, H_2_S acts as a negative regulator to control the activation of autophagy through reversible persulfidation modification to maintain the metabolic homeostasis of plant under normal or stress conditions (Figure 1). Iqbal et al. showed that H_2_S played a protective role by activating autophagy in animals [92]. However, no matter what kind of system, the regulation of autophagy through persulfidation seems to have a common purpose, that is, to protect survival.

## 5. Protein Persulfidation and *S*-Nitrosylation

Maintaining the homeostasis of the intracellular environment involves a very complex process, which is regulated by a multi-level signal network, especially during stress. In addition to the previously mentioned ROS, RNS is also a class of important signal molecule when plants adapt to adversity. Among them, the function and regulation mechanism of NO in plants have been extensively studied in recent decades. As a signaling molecule, NO involves almost all stages of a plant’s entire lifespan. In the initial stage, some researchers used pharmacological or biochemical methods to prove that NO plays an important protective role in plant disease resistance and abiotic stress [93,94]. However, the molecular mechanism of NO in plants has received widespread attention in recent years. Emerging data suggest that NO exerts its regulatory role through a variety of distinct mechanisms [95]. In particular, just like persulfidation induced by H_2_S, NO could induce *S*-nitrosylation, which was a reversible PTM and could inhibit or activate protein functions [96]. Shi et al. showed that both NO and H_2_S could improve the cadmium tolerance of bermudagrass [97]. Intriguingly, both NO and H_2_S scavengers or inhibitors could block NO signaling, but H_2_S signaling could only be abolished by H_2_S specific inhibitors or scavengers. In tomatoes, Cristiane et al. found that H_2_S started to accumulate after NO accumulated to a certain amount under high salinity stress, and this seemed to be an established procedure [98]. At the same time, the transcript level of H_2_S synthesis-related enzyme genes enhanced with the accumulation of NO. In addition, Iqbal et al. reported that exogenous NO and H_2_S could alleviate the suppression of wheat photosynthesis caused by high temperature by enhancing antioxidant system and ascorbate-glutathione metabolism [99]. However, in this process, the scavenger of H_2_S weakened the NO-mediated mitigation effect. Therefore, there is an inevitable connection between H_2_S and NO in the process of plant stress response. However, it has not been reported whether there is a certain relationship between persulfidation and *S*-nitrosylation.

Macroscopically, these two PTMs both occur on thiol moieties of protein cysteine residues. Indeed, it is not difficult to find that there seems to be a connection between persulfidation and *S*-nitrosylation based on the existing evidence. Used a site-specific nitrosoproteomic method, Hu et al. identified 926 proteins that could undergo *S*-nitrosylation modification in *A. thaliana*, but only a few of these proteins have known specific functions [100]. The same is true for current research on persulfidation. Compared the newly identified persulfidated and nitrosylated proteins in *A. thaliana* and found that the number of proteins that have undergone persulfidation modification is greater than that of *S*-nitrosylation proteins. Therefore, the possible result was that the persulfidation modification might be more important than *S*-nitrosylation modification in plants. Moreover, there were 639 proteins that could be both persulfidated and nitrosylated. María et al. showed that the content of H_2_S and NO in sweet peppers increased significantly during the mature period [101]. Further found that NO could induce the *S*-nitrosylation of Cys75 of NADP-isocitrate dehydrogenase (NADP-ICDH), a NADPH regenerating enzyme, and thereby inhibiting NADP-ICDH enzyme activity. Similarly, H_2_S could also inhibit activity of NADP-ICDH. In fact, it has been confirmed that NADP-ICDH was a persulfidation target in mammals [102]. Due to the high degree homology of this enzyme in different species, the authors speculated that the inhibitory role of H_2_S on NADP-ICDH was achieved through persulfidation modification in plants (Table 2), but the specific mechanism needs to be explained by experimental evidence.

We previously discussed that SnRK2.6 could undergo persulfidation modification at Cys131 and Cys137. Strikingly, Wang et al. showed that Cys137 of SnRK2.6 could also undergo *S*-nitrosylation modification and negatively regulate SnRK2.6 activity in *A. thaliana* under drought stress [106] (Table 2). Likewise, the Cys890 site of RBOHD can also be modified by both persulfidation and *S*-nitrosylation (Table 2), but these two PTMs have opposite regulatory effects on RBOHD. It is not difficult to infer that these two PTMs caused by H_2_S and NO have a competitive relationship in plants under certain conditions. In addition, current evidence suggests that some redox-related enzyme activities could be regulated by two PTMs under normal or stressful conditions. *S*-nitrosylation modification reduced the activity of CAT, GAPDH, and increased the activity of APX [103,104,105,107]. Yet, as mentioned earlier, the persulfidation modification improved the activity of APX, GapC1, and reduced the activity of CAT (Table 2). Therefore, the two different PTMs, persulfidation and *S*-nitrosylation, may synergistically or competitively regulate the activity of certain enzymes to maintain the metabolic balance of the plant system (Figure 1).

Apart from the common regulation of these enzymes, Serrato et al. showed that Cys153 *S*-nitrosylation of fructose-1,6-bisphosphatase (FBPase), a key Calvin-Benson cycle (CBC) composing enzyme, could trigger the formation of Cys153-Cys173 disulfide bond in FBPase in *Pisum sativum* [108]. Therefore, *S*-nitrosation may be one of the prerequisites for the formation of persulfidation. In line with the previously mentioned, H_2_S is regulated by NO. We speculate that the formation of persulfidation may be based on *S*-nitrosation. Although there are various signs that persulfidation and *S*-nitrosylation are inevitable during the growth and development and resistance of plants, the clear crosstalk needs further experimental data to explain.

## 6. Conclusions and Future Perspectives

In the past few decades, the effects of sulfide on the growth and development of plants and the maintenance of metabolic balance have been widely discussed. Among them, H_2_S mediates a series of plant growth and development processes and adversity responses, and protein persulfidation has recently been widely recognized as the main pathway through which H_2_S exerts its biological effects. Therefore, the identification and functional analysis of persulfidation proteins in plants will not only contribute to understand the molecular mechanism based on H_2_S, but also in further investigate the role of protein persulfidation in the plant signal network. Based on the above discussion, protein persulfidation has important biological significance in at least two aspects in plant systems. On the one hand, as H_2_S accumulates, it will not cause a global response of the proteome containing persulfidation sites, but prefer to induce the persulfidation of the target protein in an independent manner under a given stimulus. The H_2_S production/depletion and H_2_S targets for persulfidation might be strictly controlled by other signals and spatially or temporally separated. On the other hand, protein persulfidation is involved in important metabolic pathways and signaling. For example, one of which can arouse waves of plant antioxidant system by modifying part of the protein in the antioxidant system and changing the activity of antioxidant enzymes. Another is associated with phytohormone networks, especially refers to ABA and ETH, as a considerable part of the proteins in the ABA and ETH signaling pathways undergo persulfidation. Moreover, although the advantages and disadvantages of autophagy in plants have not been clearly defined, it is currently believed that the persulfidation in the autophagy pathway plays a negative regulatory role. However, regardless of the signal pathway, protein persulfidation seems to act as a protective mechanism in plants.

Although the research of persulfidation in plants has made some progress, there are still very few persulfidation proteins whose functions have been specifically analyzed. Since the redox homeostasis maintains a high dynamic balance in the cell, it poses a huge challenge for the detection of redox-based persulfidation proteins. To further complicate this picture, for a given protein, there may be more than one site that can undergo persulfidation and the differences between these modification sites may result in changes in protein activity, location, stability, and ability to interact with other proteins. In order to better understand the role of protein persulfidation in plants, it is necessary to continue to explore the selective mechanism of persulfidation modification on the target protein, find the master proteins and sites in the persulfidation regulation signal pathway, reveal the interaction between persulfidation and other PTMs, and improve the detection method of persulfidation protein. Additionally, it will also be beneficial to explore the changes in protein structure and the formation of active domains caused by persulfidation. All these complexities often necessitate detailed case study methods, which will bring certain challenges to the research in this field. Therefore, investigating the role of protein persulfidation in plants is a very promising field.

## Figures and Tables

**Table 1:** Effect of persulfidation proteins on the activity and come-off in the abiotic stress of plants.

Metabolic Process	Plant Species	Abiotic Stress	Protein Modified	Sites	Protein Activity	Functions	Reference
Anti-oxidant system	*Solanum lycopersicum*	CuO NPs	CAT1	Cys234	↓	Anti-oxidation	[7]
			APX1	Cys168	↑		
			POD5	Cys46, Cys61	↑		
	*Arabidopsis thaliana*	Cold	MPK4		↑	Enhances the signal cascade of MAPKs, thereby alleviating stress	[8]
ABA signaling	*A. thaliana*	Drought	DES1	Cys44, Cys205	↑	Promotes the release of H_2_S from DES1	[59]
	*A. thaliana*	Drought	RBOHD	Cys825, Cys890	↑	Improves the ability of RBOHD to generate ROS	[59]
	*A. thaliana*	Drought	SnRK2.6	Cys131, Cys137	↑	Strengthens the interplay between SnRK2.6 and ABF2, therefore promotes ABA signaling	[59]
	*A. thaliana*	Drought	ABI4	Cys250	↑	Enhances the transcription of MAPKKK18, therefore promote MAPK cascade	[9]
ETH signaling	*S. lycopersicum*	Osmotic	LeACO1	Cys60	↓	Inhibits the ETH synthesis	[10]
	*S. lycopersicum*	Osmotic	LeACO2		↓	Inhibits the ETH synthesis	[10]
Autophagy pathway	*A. thaliana*	Nitrogen starvation	ATG4a	Cys170	↓	Weakens the catalytic effect of ATG8 and PE conjunction, therefore inhibits autophagy	[60]
	*A. thaliana*	ER-phagy	ATG18a	Cys103	↑	Enhanced ATG18a affinity and co-localization time with phagophore membranes, thus delaying the release and maturation of autophagosomes	[61]

↑ denotes increase, ↓ denotes suppression. CuO NPs, Copper Oxide (II) nanoparticles; CAT1, catalase 1; APX1, ascorbate peroxidase 1; POD5, peroxidase 5; MPK/MAPK, mitogen-activated protein kinases; ABA, abscisic acid; DES1, DESULFHYDRASE 1; H_2_S, hydrogen sulfide; RBOHD, respiratory burst oxidase homolog protein D; ROS, reactive oxygen species; SnRK2.6, SNF1-RELATED PROTEIN KINASE2.6; ABF2, ABA RESPONSE ELEMENT-BINDING FACTOR2; ABI4, ABSCISIC ACID INSENSITIVE 4; MAPKKK18, Mitogen-Activated Protein Kinase Kinase Kinase 18; LeACO1/ LeACO_2_, ACC oxidases; ETH, ethylene; ATG, autophagy-related proteins; ER-phagy, endoplasmic reticulum autophagy.

**Table 2:** Various Proteins are persulfidated and *S-*nitrosylated in plants.

Plant Species	Protein Modified	Persulfidation/ *S*-nitrosation Sites	Persulfidated/ *S*-nitrosated Activity	Reference
*Capsicum annuum* L.	NADP-ICDH	--/ Cys75	↓/↓	[101]
*Arabidopsis thaliana*	SnRK2.6	Cys131, Cys137/ Cys137	↑/↓	[68,100]
*A. thaliana*	RBOHD	Cys825, Cys890/ Cys890	↑/↓	[59,103]
*Solanum lycopersicum* * A. thaliana ^#^	CAT1	Cys234/--	↓/↓	[7,104]
*S. lycopersicum* * Pisum sativum L. ^#^	APX1	Cys168/ Cys32	↑/↑	[7,105]
*A. thaliana*	GapC1	Cys156/ Cys160	↑/↓	[41]

↑ denotes increase, ↓ denotes suppression, -- denotes unclear, * denotes persulfidated plant species, **#** denotes *S*-nitrosylated plant species. NADP-ICDH, NADP-isocitrate dehydrogenase; SnRK2.6, SNF1-RELATED PROTEIN KINASE2.6; RBOHD, respiratory burst oxidase homolog protein D; CAT1, catalase1; APX1, ascorbate peroxidase1, GapC1, cytosolic glyceraldehyde-3-phosphate dehydrogenase.

## Supplementary Materials

The following are available online at https://www.mdpi.com/article/10.3390/antiox10101631/s1, Table S1: The biosynthesis and degradation of H_2_S in animal and plant cells
