# Supplementary material for: Protein Persulfidation in Plants: Function and Mechanism
Source: Antioxidants (Basel). 2021 Oct 16;10(10):1631. doi: 10.3390/antiox10101631 (PMC8533255; doi:10.3390/antiox10101631)
Supplement: Supplementary file 1 [file antioxidants-10-01631-s001.zip › antioxidants-1398221-supplementary.pdf]

Supplementary Table S1. The biosynthesis and degradation of H_2S in animal and plant cells

	**Synthetase**	**Synthetase Substrate**	**Degrading enzyme**	**References**
Animals	Cystathionine γ-lyase (CSE)	L-cysteine	Sulfide: quinone reductase/ Sulfur dioxygenase/ Thiosulfate: cyanide sulfur-transferase	[17]
	Cystathionine β-synthase (CBS)	L-cysteine and L-homocysteine		
	3-mercaptopyruvate sulfur transferase	Thioredoxin (Trx) and dihydrolipoic acid (DHLA)		
Plants	Desulfhydrase L-Cys (L-CD)	L-cysteine	OAS-TL	[22]
	D-cysteine Desulfhydrase (D-CD)	D-cysteine		
	Cyano alanine synthase (CAS)	Cyanide		
	Sulfte reductase (SiR)	Sulfate		
	O-acetylserine-thiol lyases (OAS-TL)	Cysteine		[25]
